# The structure of creative cognition in the human brain

**DOI:** 10.3389/fnhum.2013.00330

**Published:** 2013-07-08

**Authors:** Rex E. Jung, Brittany S. Mead, Jessica Carrasco, Ranee A. Flores

**Affiliations:** Department of Neurosurgery, University of New MexicoAlbuquerque, NM, USA

**Keywords:** creativity, default mode network, blind variation, divergent thinking, structural neuroimaging, magnetic resonance spectroscopy, diffusion tensor imaging

## Abstract

Creativity is a vast construct, seemingly intractable to scientific inquiry—perhaps due to the vague concepts applied to the field of research. One attempt to limit the purview of creative cognition formulates the construct in terms of evolutionary constraints, namely that of blind variation and selective retention (BVSR). Behaviorally, one can limit the “blind variation” component to *idea generation* tests as manifested by measures of divergent thinking. The “selective retention” component can be represented by measures of convergent thinking, as represented by measures of remote associates. We summarize results from measures of creative cognition, correlated with structural neuroimaging measures including structural magnetic resonance imaging (sMRI), diffusion tensor imaging (DTI), and proton magnetic resonance spectroscopy (1H-MRS). We also review lesion studies, considered to be the “gold standard” of brain-behavioral studies. What emerges is a picture consistent with theories of disinhibitory brain features subserving creative cognition, as described previously (Martindale, 1981). We provide a perspective, involving aspects of the default mode network (DMN), which might provide a “first approximation” regarding how creative cognition might map on to the human brain.

## Definitions

Creativity is a complex and vast construct that has been vital to the progress of human civilization and very likely the development of human reasoning processes. Indeed, the immense array of creative endeavors encompasses the works of such disparate activities as those undertaken by painters, sculptors, nuclear engineers, landscape architects, graphic designers, and software developers: how do we imagine to capture such a broad construct? At the onset there should be noted two major potential pitfalls for creativity/neuroimaging research: the singular focus on the iconic genius—known as Big “C”—at the expense of the vast majority of creative endeavors undertaken by the other 99% of the distribution of creative endeavors—known as little “*c*” (Stein, 1953), and undue focus on an encompassing definition around which largely unedifying academic arguments often ensue (e.g., “gene” has no commonly accepted definition although research in this area progresses apace; the same can be said for cognitive constructs such as “intelligence” and “creativity”) (Arden et al., 2010). To be sure, much of value can be learned from the historiometric assessment of great giants of creative history (think Mozart, Einstein, Van Gogh—the list goes on), divining how they might have lived, what formative experiences they might have had, what their neurological makeup might have looked like (Simonton, 1984); unfortunately, these individuals and their magnificent brains are (save Einstein) lost to history. Just as fortunate, individuals who make up the vast underbelly of the “*c*” portion of the distribution avail themselves to us to this day, indeed offer themselves readily to most neuroimaging experiments (in exchange only for some nominal compensation, a.k.a. “beer money”).

With these and several other well articulated caveats in mind (Dietrich, 2007), any truly plausible definition of creativity, intelligence, or other broad behavioral construct must be applicable not just to humans, and not just to exceptionally talented humans (i.e., “genius”), but also to other species and across evolutionary time. Thus, for the purposes of this neuroscience of creativity discussion, we adopt a broadly accepted definition of creativity, which refers to *the production of something both novel and useful* (Stein, 1953; Martindale, 1999; Runco and Jaeger, 2012). This definition is plausible, is broadly applicable, and would appear to hold true across much of evolutionary time. As such, it also refers to the workings of the brain.

## Creativity as blind variation and selective retention

While the varieties of creative expression are many (i.e., domain specific), the cognitive processes critical to its manifestation (i.e., domain general) are likely to be relatively few; thus, in order to make the problem tractable, researchers have attempted to identify cognitive processes central to creative cognition. In 1960, Donald Campbell attempted to explain the development of creative thought with a theory of “blind variation and selective retention” (BVSR). Campbell presents the process of “achieving innovation” as the next step in the evolutionary progression from blind floundering to an intelligent knowledge process (Campbell, 1960). Campbell notes similarities between “trial-and-error” problem solving and natural selection in evolution, namely “a mechanism for introducing variation, a consistent selection process, and a mechanism for preserving and reproducing the selected variations (p. 381).” The emphasis on “blind” as opposed to “random” is important, as the variations are seen to be independent of the environmental conditions from which they might have sprung. This simple law states that “the greater the heterogeneity and volume of trials the greater the chance of a productive innovation (p. 395).” This law has been codified by Dean Keith Simonton, who provides extensive and compelling support for such a BVSR system underlying creative cognition (Simonton, 1999). Critiques to the notion of BVSR underlying creative cognition have also been raised (Gabora, 2011). The blind-variation component reflects elements of divergent thinking measures (e.g., tell me as many ways you can think of to use a brick) insofar as it hinges on the ability to generate a large number of novel ideas. Simultaneously, as scores of exploratory thought trials are filtered through the mind, the selection criteria are eventually met and the innovative process is terminated. This ability to eliminate the absurd and frivolous from the meaningful and appropriate makes up the “selective retention” component of Campbell's theory, and is also measured by the usefulness or appropriateness of the new use for the common item (e.g., BRICK = to grind corn into meal) (Campbell, 1960). Criticism of the BVSR theory rely on its potential lack of falsifiability (Simonton, 2010), although it stands as a compelling model for cognitive processes underlying creativity.

## Neuroimaging studies of creativity

The majority of psychometric research studies in creativity have emerged in the latter half of the 20^th^ century (Guilford, 1968; Torrance, 1974; Amabile, 1982), but little progress has been made regarding brain correlates of this construct prior to the advent of modern neuroimaging techniques. Whereas, neuroimaging studies of intelligence have a 20-year history and span dozens of studies (Jung and Haier, 2007), similar studies of creativity are relatively few although spanning roughly the same period of time. Neuroimaging of the creative process can be undertaken to assess brain traits [structural magnetic resonance imaging (sMRI); diffusion tensor imaging (DTI); proton magnetic resonance spectroscopy] and brain states (functional Magnetic Resonance Imaging; Magnetoencephalography; Electroencephalography) associated with task performance. Both the behavioral and neuroimaging approaches can be combined to select people as high and low on trait measures of creativity and then compare the state of their brain functioning as they perform creative tasks. For example, imaging studies of intelligence have identified a network of areas where intelligence test scores correlate to brain features; these areas are distributed throughout the brain but most prominent in parietal and frontal areas (Haier and Jung, 2007; Jung and Haier, 2007). Another approach is to image the state of brain function as it fluctuates in people performing creative tasks. Of course, the field is not sufficiently well developed to have focused specifically upon subcomponents of creative cognition, although some studies do distinguish between measures of convergent vs. divergent reasoning (Fink and Neubauer, 2006), insight (Jung-Beeman et al., 2004), implicit thought processes (Haider, 1992; Kaufman et al., 2010), and other promising candidates, most recently those of “conceptual expansion” and “constraints of examples” (analogous to “BV” and “SR” respectively of BVSR) (Abraham and Windmann, 2007).

Methodologically, it is likely impossible to capture someone being truly creative in a laboratory setting; rather, we describe ways by which to measure this cognitive construct by capturing particular elements found to be important to the creative process including, “insight,” “convergent,” and “divergent” cognitive processes. Divergent tasks are characterized by having many possible answers as opposed to having one correct answer (i.e., “convergent thinking”) characteristic of most measures of intelligence and reasoning. We also describe a “Consensual Assessment Technique” (Amabile, 1982) by which independent judges might rank the creative products of each subject, with high inter-rater reliability, from which a “composite creativity score” can be compiled. Following intelligence studies, one approach to creativity research is to use neuroimaging to identify brain features (structural and functional) which differ between individuals deemed as being high or low on a trait of creativity as assessed by various measures (e.g., psychometric tests, peer evaluations). We focus on structural measures below.

## Why structural studies?

One of the tasks facing research in the field of creativity is the difficulty in measuring such a complex entity. Proxy measures such as divergent thinking tasks have been heavily relied upon in the laboratory, though they are at best a measure of creative potential and cannot assess lifetime creative output or impact of creative products (Piffer, 2012). For this reason, the neurosciences have begun to look for highly reliable and valid ways to measure creative cognition. It is essential to any scientific endeavor that reproducible results are obtained so that new information can be effectively shared with the scientific community, thus to build upon the foundation of scientific knowledge. Without reliable results, unmeasured error can be incorporated into the data set and ultimately hinder the progress of scientific knowledge (Bennett and Miller, 2010). While there are a growing number of neuroimaging techniques available for research on creativity, this paper limits its scope to highly reliable and reproducible test methods and results. The essence of this review is to summarize the best results this field has to offer from sMRI, DTI, and proton magnetic resonance imaging (1H-MRS). In addition, the results of lesion studies, considered the “gold standard” of brain-behavioral studies, are discussed.

First, morphometric measures were used to analyze correlations between cortical thickness and creative achievement. Wonderlick et al. showed that surface maps of cortical thickness were highly reproducible with Intra-class correlation analyses ≥0.95. More recently, side-by-side comparisons of the three volumetric segmentation algorithms (Voxel Base Morphometry, FreeSurfer, and FAST) found extremely high reliability for the first two algorithms (≥0.99), with FAST being ≥0.90, with all segmentation techniques tending to underestimate gray matter volume (Eggert et al., 2012). Second, spectroscopic studies are presented to demonstrate the relationship between laboratory measures of creativity and the concentrations of N-acetyl-aspartate (NAA), a biomarker for neuronal integrity. In a study conducted by Gasparovic et al. to assess the test–retest reliability and reproducibility of ^1^H magnetic resonance spectroscopic imaging (^1^H-MRSI), the tissue-specific estimates of NAA metabolite were obtained with high reliability and reproducibility with interclass correlation coefficient (ICC) values ≥0.90 (Gasparovic et al., 2011). DTI was utilized to assess whether white matter integrity, and structural connectivity, measured using fractional anisotrophy (FA), was related to composite creativity scores (Jung et al., 2010a). Danielian et al. showed that fiber tracking measurement has excellent inter-rater reliability and test–retest precision demonstrating ICCs ≥0.77 for all evaluated tracts (Danielian et al., 2010). The results from these studies indicate that the test measures discussed in this review are highly accurate and impactful.

## Where do we begin looking in the brain for sources of creative cognition?

Neurological inquiries regarding creativity have tended to focus upon whether the frontal lobes are engaged or whether more posterior brain regions (Heilman et al., 2003) or subcortical structures such as the basal ganglia are more predominant (Dietrich, 2004; Flaherty, 2005). From these myriad perspectives have emerged several attempts designed to capture the neuroscience of creativity, based largely on data gleaned from neurological and psychiatric patients and largely confined to artistic expression (Pollack et al., 2007). Indeed, *de novo* artistic expression have been associated with left fronto-temporal (Finkelstein et al., 1991) and right temporal lobe epilepsy (Mendez, 2005), several case studies of fronto-temporal lobe dementia (FTLD) (Miller et al., 1998, 2000; Thomas Anterion et al., 2002), a case of Parkinson's disease treated with dopaminergic agonists (Schrag and Trimble, 2001), and a single case of subarachnoid hemorrhage (Lythgoe et al., 2005). Miller postulates that the selective atrophy of the anterior temporal and basal frontal lobes that accompanies FTD may reduce inhibition of the more posteriorly located visual systems, resulting in the patients' heightened interest in artistic works (Miller et al., 1998). Similarly, in a patient with primary progressive aphasia, a profound increase in artistic interest and ability coincided with significant atrophy of the left inferolateral frontal cortex (Seeley et al., 2008). However, subsequent systematic study of artistic ability associated with the various dementias found no general increase in creativity to be linked with fronto-temporal dementia (or semantic or dementia of the Alzheimer's type), with the authors noting that “despite the existence of these isolated patients with increased artistic production, however, apathy leading to diminished creativity is more clinically typical of patients with FTLD, suggesting that these case studies may be the exception rather than the rule (Rankin et al., 2007).

In contrast to fronto-temporal degenerative *facilitation* of artistic creativity, other lesion studies have indicated that certain parietal lesions can lead to *reduced* creative ability, at least within the visual arts. Lewy Body Dementia is a disease that is characterized by progressive degeneration of visuo-spatial skills and constructional abilities. In a case study presented by Drago et al., a 78-year-old visual artist experienced gradual reduction in his ability to express his artistic subject matter. This loss of expression was attributed to cellular deterioration of the parietal lobes. Throughout the progression of his disease, the artist preserved the ability to create novel works of art, which is proposed to coincide with preserved frontal lobe function. This case study seems to provide support for the importance of visuospatial cortical networks in artistic creation and ultimately the parietal lobes (Drago et al., 2006).

What these disparate lesions have to say about the creative process, particularly as related to creative cognition, is hard to say other than to speculate regarding the likely *disinhibitory* nature of lesions located within an eloquent *network* producing *increased* behavioral output. For example, Flaherty notes that the temporal lobes modulate creative drive, but notes also that changes to the temporal lobes characterize other neurological syndromes including hypergraphia, pressured speech, hypomania, and even hallucinations (p. 149, Table 1). She further states that: “to a first approximation” the corticocortical connections between frontal and temporal lobes are “mutually inhibitory” (p. 149, Figure 1) (Flaherty, 2005). We interpret this to imply that, with regard to a possible neurological framework underlying creativity, we must look not only to *increased* neural tissue in key brain regions, but perhaps also to some mismatch between mutually excitatory and inhibitory brain regions that form a network subserving such complex human behaviors as preparation, incubation, illumination, and verification components of creative cognition. This notion of a delicate interplay of both *increases* and *decreases* in neural mass, white matter organization, biochemical composition, and even functional activations within and between brain lobes and hemispheres is an important notion, critical to a full understanding of the neurological underpinnings of creative cognition.

**Table 1:** **Structural studies reviewed**.

**Author (date)**	***N***	**Proxy test measures**	**Higher brain integrity-higher creativity**	**Lower brain integrity-higher creativity**
**MORPHOMETRY STUDIES**				
Jung et al. (2010a,b,c)	61	Three divergent thinking tasks: design fluency test, four line condition of the DFT, uses of objects test	R. posterior cingulate	L. lingual gyrus, R. fusiform, R. cuneus, R. angular gyrus, R. vertices form inferior parietal, superior parietal and lateral occipital
		Creative achievement test	R. angular gyrus	L. lateral orbitofrontal
Takeuchi et al. (2010a,b)	55	S-A creativity test	Regional gray matter volume: R. dorsolateral prefrontal cortex, bilateral striate, a cluster that includes the dorsal midbrain, the reticular formation, the periaqueductal gray, the ventral midbrain (substantia nigra and ventral tegmental area), and regions in the precuneus	
		Raven's advanced progressive matrix		
Gansler et al. (2011)	18	Torrance test of creative thinking	R. parietal lobe	Corpus callosum area (splenium region)
**SPECTROSCOPY STUDIES**				
Jung et al. (2009a,b)	56	Three divergent thinking tasks: design fluency test, four line condition of the DFT, uses of objects test	L. anterior gray matter NAA	R. anterior gray matter NAA
		Controlled oral word association test (COWAT)		
		Wechsler abbreviated scale of intelligence (WASI)		
		NEO factor five inventory: neuroticism, extraversion, openness, agreeableness, and conscientiousness		
**DIFFUSION TENSOR IMAGING**				
Jung et al. (2010a,b,c)	72	Four divergent thinking tasks: verbal and drawing creativity tests, four line condition of the DFT, uses of objects test, and generation of captions to a New Yorker Magazine cartoon		FA within predominantly left inferior frontal white matter (i.e., regions overlapping the uncinate fasciculus and anterior thalamic radiation)
		Wechsler abbreviated scale of intelligence (WASI)		
		NEO factor five inventory: neuroticism, extraversion, openness, agreeableness, and conscientiousness		FA within the right frontal white matter (i.e., regions overlapping the uncinate fasciculus and anterior thalamic radiation)
Takeuchi et al. (2010a,b)	55	S-A creativity test	Frontal lobe, anterior cingulate cortex bilaterally extending into the body of the corpus callosum, white matter regions adjacent to the anterior part of the bilateral inferior parietal lobe and a white matter region extending into the right temporo-parietal junction from the frontal lobe (arcuate fasciculus) and the right occipital lobe	
		Raven's advanced progressive matrix		
**LESION STUDIES**				
Shamay-Tsoory et al. (2011)	Medial prefrontal cortex lesion (mPFC) *N* = 12	Neuropsychological assessment, torrance test of creative thinking, alternate uses test	R. mPFC lesions were associated with impaired originality	
	Inferior frontal gyrus lesion (IFG) *N* = 7			L. IFG lesions exhibited high originality scores
	mPFC and IFG lesions *N* = 6			
	Posterior lesions (PC) involving damage in the temporoparietal, inferior parietal, or superior parietal lobule *N* = 15			Positive correlation between lesions in the left PC and originality scores
Abraham et al. (2012b)	Frontal lobe: frontal lobe extensive (FL-EXT), frontal lobe lateral (FL-LAT), frontal lobe polor and/or orbital (FL-ORB). *N* = 29	Torrance test of creative thinking, alternate uses test	Poor performance on fluency, originality and creative imagery	FL-POL performed better on constraints of examples tests
	Basal ganglia (BG) *N* = 16		Poor performance on originality, practicality and incremental problem solving	Superior performance on the constraints of examples test
	Parietal-temporal lobe (PTL) *N* = 11		Poor Performance on fluency, practicality and constraints of examples	

*R., right hemisphere; L., left hemisphere; DFT, design fluency test; NAA, N-acetylaspartate; COWAT, controlled oral word association test; WASI, Wechsler abbreviated scale of intelligence; FA, fractional anisotropy; mPFC, medial prefrontal cortex; IFG, inferior frontal gyrus; PC, posterior cortex; FL-EXT, frontal lobe - extensive; FL-LAT, frontal lobe - lateral; FL-ORB, frontal lobe - orbital; FL-POL, frontal lobe - polar; BG, basal ganglia; PTL, parietal-temporal lobe*.

**Figure 1:**
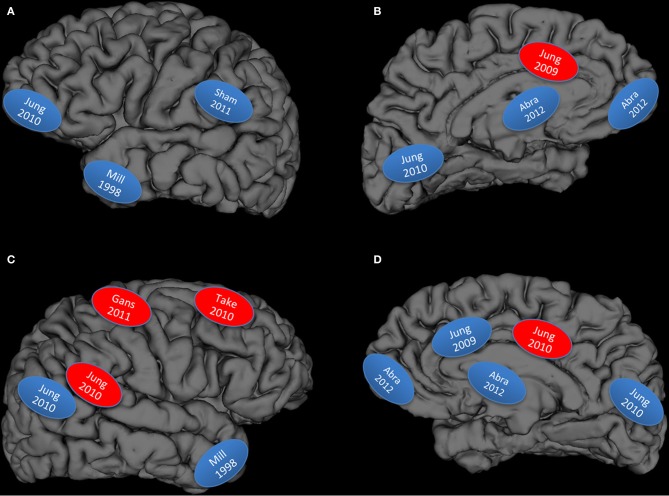
**Graphical display of studies reviewed: Blue, lower brain integrity measures associated with higher creativity measures; Red, higher brain integrity measures associated with higher creativity measures; **(A)** left lateral hemisphere; **(B)** left medial hemisphere; **(C)** right lateral hemisphere; **(D)** right medial hemisphere; Abra, Abraham et al., 2012a; Mill, Miller et al., 1998; Gans, Gansler et al., 2011; Sham, Shamay-Tsoory et al., 2011; Take, Takeuchi et al., 2010a,b**.

## Structural magnetic resonance imaging (sMRI)

The brain is not easily parceled into segmented regions in spite of the elegant cellular organization articulated by Brodmann (1905). Indeed, the accurate measurement of cortical and subcortical tissue volumes, thickness, and density has only been recently realized with the advent of voxel based morphometry (VBM) and extended with analysis techniques including, but not limited to, FreeSurfer (surfer.nmr.mgh.harvard.edu). VBM is a method by which standard T1 images may be automatically segmented into tissue compartments (i.e., gray, white, cerebrospinal fluid) using measures of voxel intensity at the millimeter level of resolution (Ashburner and Friston, 2000). Images from individual subjects are imported into a freely available analysis program (i.e., statistical parametric mapping, or SPM), spatially normalized in stereotactic space (i.e., Montreal Neurological Institute), segmented and smoothed, and subjected to voxel-wise statistical comparisons with either a comparison group or an external variable (Ashburner and Friston, 2000).

sMRI was utilized to hypothesize a link between the results of divergent thinking and creative achievement test and cortical thickness (Jung et al., 2010c). Subjects participating in this study were administered the creative achievement questionnaire (CAQ)^1^, an objective and reliable measure of creative productivity (Carson et al., 2005) in addition to three different divergent thinking tasks. Each subject's creative products were assessed by three independent judges and scored on a composite creativity index (CCI) (Amabile, 1982)—consensual assessment technique. Finally, sMRI was used to investigate the correlation between creativity scores and cortical thickness. Results indicated that increased gray matter thickness in the right posterior cingulate gyrus and the right angular gyrus correlated positively with higher CCI and CAQ performance respectively. Conversely, there were several areas that had a negative relationship with CCI and CAQ scores, in that decreased cortical thickness was associated with higher creativity scores. Decreased thickness within regions including the left frontal lobe, lingual, cuneus, angular, inferior parietal and fusiform gyri predicted performance on the CCI. For CAQ, there was only one area where decreased cortical thickness related to higher scores; the left lateral orbitofrontal region. These results appear to indicate that information flow between many different areas of the brain may be necessary to the development of creative ideation and achievement respectively (Jung et al., 2010c). Unlike other studies of ability (e.g., Draganski et al., 2004), the novel finding of this report was that *decreased* cortical thickness in discrete areas of the frontal and posterior cortical regions, was associated with increased creative ability.

Takeuchi et al. set out to study the relationship between regional gray matter volume (rGMV) in subcortical regions and individual creativity. All subjects (42M, 13F) were administered the S-A creativity test, designed to evaluate creativity using three DT tasks, and assigned a total creativity score (Takeuchi et al., 2010a). Moreover, the Raven's Advanced Progressive Matrix, a psychometric test of general intelligence that is well correlated with general IQ test results (Raven, 1994), was used to measure each subjects intellectual capacity. These results were compared with morphometric data collected via MRI and revealed significant, positive correlation with creativity scores in the following regions: right dorsolateral pre-frontal cortex (DLPFC), bilateral striatum, the dorsal midbrain, the reticular formation, the periaqueductal gray (PAG), the ventral midbrain (substantia nigra and ventral tegmental area) and regions in the precuneus. The authors interpret their findings of increased fGMV in the dopaminergic systems of the brain to correspond with the notion that the complex construct of creativity requires diverse cognitive abilities, such as working memory, sustained attention, cognitive flexibility and fluency in the generation of ideas. It should be noted that these results are in contrast to those reported by Jung et al., in the same year: How could this be? One explanation could be the very high predominance of male subjects in the Takeuchi sample (3M:1F) as compared to the Jung sample (~1M:1F). Given numerous previous studies showing sex differences associated with brain-behavior relationships (Yurgelun-Todd et al., 2002; Haier et al., 2005; Jung et al., 2005; Schmithorst, 2009; Wang et al., 2012), the comparability of these studies should be interpreted with caution. Other reasons could also explain the differences between the results, including different divergent thinking tasks used, different analysis methods employed, and different imaging parameters, to name a few. Future studies will help to determine more consistent relationships between white matter integrity and creativity.

Finally, Gansler et al. hypothesized that the torrance test of creative thinking (TTCT), one of the most commonly accepted methods to measure visual and verbal DT production tasks (Torrance, 1974) should be linked to cortical volume in specialized areas (Gansler et al., 2011). This cohort (18 M) was given the TTCT, to assess visuospatial creativity before their brain scans were subjected to VBM analysis. Results of their investigation showed significantly increased gray matter tissue volume in the right superior parietal lobe corresponded with higher TTCT scores. Additionally, the study showed that the splenium of the corpus callosum, responsible for connection the parietal and occipital lobes, negatively correlated with TTCT scores. This study emphasizes the importance of the visuospatial processing of the parietal lobe and its underlying white matter pathways to the generation of creative products (Gansler et al., 2011). Again, as this study included only male subjects, its generalizability should be interpreted with caution.

What these three morphological studies of creativity show is rather striking. Unlike studies of most cognitive capacities, including intelligence, where greater ability is associated with increased cortical thickness and/or volume (e.g., Draganski et al., 2004; Haier et al., 2005), creative cognition appears to be associated with both increases and decreases in cortical thickness and/or volume across a broad network of brain regions. Increases were observed in a network or regions corresponding to the midbrain, striatum, precuneus, and dorsolateral prefrontal cortex (Takeuchi et al., 2010a), superior parietal lobule (Gansler et al., 2011), and posterior cingulate and right angular gyrus (Jung et al., 2010b), while decreases were observed in the lingual, cuneus, angular, inferior parietal, fusiform gyri, and orbitofrontal cortex (Jung et al., 2010b), as well as the splenium of the corpus callosum (Gansler et al., 2011). The increases may be related to male gender as the two studies that found cortical increases associated with creative cognition were stilted toward males. Finally, there is a rather large correspondence of brain regions identified in these three studies and regions within and overlapping the default mode network (DMN) (Raichle and Snyder, 2007), including the precuneus, inferior parietal lobes, and medial/orbital frontal cortices.

## Magnetic resonance spectroscopy study

Proton magnetic resonance spectroscopy (^1^H-MRS) is an imaging technique that allows for neurochemistry of a research subject to be assessed *in vivo*. N-acetylaspartate (NAA) is a metabolite that is frequently used as a marker of neuronal integrity (Moffett et al., 2007). Studies have shown that high concentration of NAA in the brain is associated with higher cognitive function (Ross and Sachdev, 2004) and intelligence in normal subjects (Jung et al., 1999). Jung et al. used ^1^H-MRS to determine whether neurometabolites such as NAA could be used to predict creative ability (Jung et al., 2009b) as relationships had previously been demonstrated between this metabolite and intellectual capacity (Jung et al., 1999, 2005). As in the previous studies by this group, CCI and NEO-FFI (Openness) scores were obtained for each participant as proxy measures of creativity. The data set was assessed for relationships between behavioral and spectroscopic measures obtained from regions superior to the lateral ventricles, including white matter metabolites from frontal and parietal regions, and gray matter metabolites from the anterior and posterior cingulate cortices.

In support of the threshold effect, this study found differential metabolic profiles supporting performance on the CCI at verbal IQ's above and below a cutoff of 116: higher NAA within the left anterior cingulate predicted higher CCI performance in subjects with VIQ >116, while lower NAA with the right anterior cingulate predicted better CCI performance in subjects with VIQ ≤116. Previous behavioral studies have noted that below an IQ of 120, creativity and intelligence are weakly (~0.30) correlated, while above 120, the correlation approaches zero—the so-called “threshold hypothesis” (Guilford, 1967). This was the first biological support for the threshold hypothesis observed in a human cohort, and suggested different cognitive mechanisms associated with divergent thinking. In higher IQ subjects, it was hypothesized that “central (i.e., cingulate) facilitation of more refined access to discrete left hemisphere semantic networks” was predominant, while in lower IQ subjects “disinhibition of large right hemisphere semantic networks” predominated. Within the larger context of structural creativity studies, we interpret these findings to support a strong role for the anterior cingulate gyrus in “gating” the flow of information within prefrontal cortices during the “blind variation” component of creative cognition, which is well captured by measures of divergent thinking. The cingulate gyrus is part of the so-called “salience network,” which includes the anterior cingulate and insula, and is involved in a wide range of cognitive functions (e.g., initiation, motivation, goal directed behavior) (Devinsky et al., 1995). More specifically, and relevant to the current discussion, the dorsal anterior cingulate has been associated with orienting attention to the most relevant environmental stimuli involved with intra- and extra-personal events (Bressler and Menon, 2010).

## Diffusion tensor imaging studies

The relative contribution of white matter to higher cognitive functioning has remained relatively understudied compared to gray matter research linking particular cortical regions to performance. However, several lines of inquiry would suggest that the integrity of myelinated axons plays a critical role in intellectual attainment (Miller, 1994). For example, myelin thickness is correlated to axonal size (Bishop and Smith, 1964; Friede and Samorajski, 1967), and larger axonal diameter is associated with increased nerve conduction speed (Aboitiz, 1992). The simultaneous increases in myelination and axonal diameter have been hypothesized to play a critical role in cognitive development. One imaging modality particularly amenable to measurement white matter integrity is DTI, an imaging technique that measures the coherence of water movement through the white matter of the brain and that can facilitate *in vivo* white matter fiber tracking (Mori and van Zijl, 2002). Since the diffusion of water down the axon is faster than it would be in the perpendicular direction, it can be presumed that the axonal membrane and myelin sheath exist as the main barrier to perpendicular diffusion. Therefore, the diffusion is anisotropic meaning that the diffusion rates differ based on direction (Pierpaoli and Basser, 1996; Pierpaoli et al., 1996). Fractional Anisotropy (FA) is considered to be an overall measure of axonal integrity and therefore a higher FA value indicates greater axonal coherence and/or myelination of the axon. Patients with neurological disorders (e.g., multiple sclerosis, stroke) tend to show reduced FA indicating disruption of axon and myelin microstructure in the tissue (Danielian et al., 2010).

In effort to explore the relationship between creativity and the microstructure of the brain's white matter, Takeuchi et al. used DTI to determine whether white matter integrity is related to proxy measures of creativity. Similar to the morphometry study conducted by Takeuchi et al. subjects were administered the S-A creativity test, assigned a total creativity score, and given the Raven's Advanced Progressive Matrix intelligence test (Raven, 1994). Results showed that increased structural integrity and connectivity involving the frontal lobe and corpus collosum was positively and significantly correlated with higher creativity scores. Additionally, positive correlation was measured in the white matter of the bilateral striatum, the right temporal-parietal junction, the anterior part of the bilateral inferior parietal lobes and the right occipital lobe. This data indicates that white matter pathways facilitate creative thinking through “efficient integration of information” and “diverse high-level cognitive function” (Takeuchi et al., 2010b). The frontal lobe is responsible for many functions that are associated with creativity. Diverse cognitive abilities, regulated by the frontal lobe, such as working memory, sustained attention, idea generation and cognitive flexibility are vital to breaking old conventions and developing new patterns of thinking. Positive correlation between FA and the corpus callosum support the theory that interhemispheric connectivity is essential for information integration and the expansion of creative thought (Carlsson et al., 1994; Atchley et al., 1999).

Jung et al. also utilized DTI to evaluate white matter contribution to creative cognition (Jung et al., 2010a). Subjects who participated in this study took part in four DT tasks that were ranked by four independent judges and a CCI was derived. Importantly, none of the subjects suffered from any current or previous neurological or psychiatric disorder. Each subject also took the Wechsler Abbreviated Scale of Intelligence assessment and a self-administered personality test (NEO-FFI) to measure normal personality functioning and openness to experience. These results were compared to diffusion tensor images to analyze data correlations. Researchers found that in normal subjects, lower levels of FA within left inferior frontal white matter (i.e., regions overlapping the uncinate fasiculus and anterior thalamic radiation) scored higher on the CCI. Those subjects with lower levels of FA within the right frontal white matter scored higher of the measures of openness assessed in the self-administered personality test. Interestingly, schizophrenic and bipolar patients have also been shown to have decreased FA in these ROIs (Haznedar et al., 2005; Sussmann et al., 2009) suggesting that creativity may exist upon a continuum with psychopathology as other have proposed (Nettle and Clegg, 2006; Miller and Tal, 2007).

## Lesion studies

While neuroimaging studies have become increasingly vital to the analysis of neural networks involved in creativity, lesion studies have always been viewed as the “gold standard” of brain-behavioral studies in that they have the capacity to directly demonstrate which areas of the brain are central to cognitive functioning (Harlow, 1848; Broca, 1861; MacKay et al., 1998). Shamay-Tsoory et al. set out to localize creative thinking by comparing TTCT and neuropsychological test results of control patients with those of patients with localized lesions (Shamay-Tsoory et al., 2011). The results infer that an original thought process is the product of unique idea generation and the inhibition of stereotypical thinking. While lesions in the right medial prefrontal cortex (mPFC), were found to parallel profound impairment of creativity and originality measures, originality scores were higher when associated with left inferior frontal and posterior lesions. This may indicate that while the right PFC is responsible for generating unique ideas, it is in competition with those areas (specifically the left inferior frontal gyrus, left temporoparietal region and the left inferior parietal lobe) that are key to the production of language and the storage of logical, linear and automatic knowledge. This competition in healthy patients may actually inhibit the formation of creative thought. Therefore, in patients with lesions in the left language areas, the lack of inhibition of the right PFC may facilitate creative expression.

Abraham et al. studied neurological patients having suffered various strokes to determine the impact of lesion location on creative performance (Abraham et al., 2012a). Patients with lesions in the frontal lobe, basal ganglia and parietal-temporal lobes were matched with controls and administered standard creativity tasks (e.g., Alternate Uses Task, Remote Associates Test) as well as measures of specific aspects of creative cognition (e.g., conceptual expansion, originality, practicality, insight). The frontal lobe was further divided into subgroups to investigate the specific roles of the sections of the frontal lobe in creative thought: extensive frontal lesions (EXT), lateral frontal lesions (LAT) and frontopolar and orbital lesions (POL) (Abraham et al., 2012a). From the alternate uses test, the authors found that the LAT patients were the only ones to perform significantly worse on measures of originality and fluency. This data suggests that the frontolateral portion of the frontal lobe is specifically involved in the generation of both novel and appropriate creative responses. Parietal-temporal lobe lesions were associated with lower alternate uses fluency, lower constraint of examples practicality, and poorer performance on the constraints of examples test (i.e., inhibiting prepotent responses when given examples of divergent thinking items) (Finke et al., 1996). Basal ganglia lesions were associated with lower alternate uses originality, lower constraint of examples practicality, and lower incremental problem solving ability. This finding fits well with the literature postulated a primary function of the basal ganglia in inhibitory control (Aron et al., 2007). Interestingly, the basal ganglia group performed better on the constraints of examples test (i.e., they were able to suppress the example given) better than controls. This may indicate that the inattention and distractibility often associated with basal ganglia lesion provide an advantage when performing these tests. Increased distractibility may allow this group of patients to divert their attention past the constraint posed by salient information and increase their performance. A similar study, conducted with chronic schizophrenia patients, a disease characterized by disorganization of semantic thought, showed a positive correlation between the degree of thought disorder symptoms exhibited by this sample and performance on the constraints of examples task (Abraham et al., 2007). Finally, the POL group performed better than controls on the constraints of examples task. This finding is in contrast to the Shamay-Tsoory et al. finding that lesion of the mPFC was anti-correlated with creativity scores, although it should be noted that the discrepancies may be due to the differences in lesion overlap and/or the very different cognitive constructs tapped by the measures of “creativity” across studies (Abraham et al., 2012a).

## What does it all mean?

There has been a relatively long effort (~50 years) to localize creative processes within the human brain. Early work by Sperry and Gazzaniga with split brain patients (patients undergoing surgery to section the corpus callosum and commissures) demonstrated that the left and right hemispheres of the brain functioned independently from one another, and that “interaction through the commissures may have some particular importance for artistic drawing in the normal brain” (p. 136)—one of the many progenitors, no doubt, for the erroneous “right brain” locus of creativity (Gazzaniga and Sperry, 1967). Certainly the frontal lobe has garnered significant attention, with theories postulating fronto-subcortical modulation of catecholamines (Heilman et al., 2003), fronto-temporal mismatch producing increased creative drive (Flaherty, 2005, 2011), to the rather nebulous notion that “creativity is not particularly associated with any single brain region, the prefrontal cortex excluded” (Dietrich and Kanso, 2010). Then, there are inferences that certain aspects of creativity (e.g., insight, creative achievement) might be amenable to precise localization (Jung-Beeman et al., 2004; Jung et al., 2010c). These notions are all unlikely, as the brain does not carry out cognitive function by means of neuronal-axonal activity in discrete zones, lobes, or even hemispheres.

The brain does appear, however, to function in a manner consistent with the notion of “networks” or hubs (Buckner et al., 2009; Bressler and Menon, 2010), and this conceptualization is likely to yield more fruit in terms of brain-behavior associations with regard to creative cognition. Indeed, the brain is organized in such a way (e.g., lobes) that optimization occurs for different types of information processing (e.g., visual-occipital, auditory-temporal, sensory-parietal), with heteromodal association cortices binding together sensory information converging from multiple sources (Mesulam, 1998). More recently, it was discovered that a small number of regions within the brain, called “hubs,” possessed disproportionately large numbers of connections to other brain regions (Sporns et al., 2007), thus serving to optimize brain connectivity, and thus information transfer, across long distances (Bassett and Bullmore, 2006). These hubs can even be seen to form “networks” of brain regions corresponding to stimulus-independent thought (i.e., DMN), stimulus-dependent thought [i.e., cognitive control network (CCN)], and switching of attention between salient environmental stimuli (i.e., salience network) (Bressler and Menon, 2010). By applying the notion of “hubs” and “networks” to target cognitive processes neuroscientists within the creativity field are far more likely to link target constructs (e.g., BVSR) to specific measures (e.g., conceptual expansion) critically dependent upon fundamental neural networks (e.g., semantic network) (Kroger et al., 2012).

In his delightful book “The Art of Thought” Graham Wallace (p. 42) heralds a critical notion of creative cognition in the following passage: “The cortex of the upper brain may, for instance, of its own initiative, to satisfy its own need of activity, and to carry out its own function in the organism as a whole, start the process of thought without waiting for the primitive stimulus of sensation” (Wallace, 1931). The importance and role of “task unrelated thoughts” (Giambra, 1989) in reasoning and cognition have been the source of wonder and introspection for centuries among philosophers and, more recently, psychologists. Only very recently have neuroscientists discovered brain mechanisms that appear to underlie such “task unrelated thoughts” namely the DMN, which appear to correspond with “stimulus-independent thought” (McGuire et al., 1996). Indeed, the DMN has been associated with a broad range of task unrelated thoughts including: “remembering the past, envisioning future events, and considering the thoughts and perspectives of other people” (Buckner et al., 2008). For this reason, we hypothesize that the DMN is a good “first approximation” of a network that would serve well the purpose of BVSR in service of creative cognition. Indeed, this DMN cognitive “system” provides two of the three necessary conditions stipulated by Campbell in his BVSR conceptualization, namely: (1) “a mechanism for introducing variation,” and (2) “a mechanism for preserving and reproducing the selected variations” (p. 381), both achieved through mental manipulation and simulation (Campbell, 1960). Dean Keith Simonton has further refined Campbell's notion of BVSR, with the added notion of *sequential BVSR*, with Exploratory and Eliminatory aspects (Simonton, 2013). This type of sequential back-and-forth which begins with “informed guesses” and progresses to increasingly probable solutions can best be simulated within a network of brain regions not devoted to ongoing cognition serving environmental demands: namely the DMN.

But how well does the data fit the hypothesis? We have rendered Figure 1 to summarize the results from the structural and lesion studies reviewed above: what emerges is a figure that begins to resemble large scale cortical networks, particularly the DMN and associated hubs (Buckner et al., 2008; Bressler and Menon, 2010). However the fit is not perfect, as would be expected given that the DMN is a large network involved in “constructing dynamic mental simulations based on personal past experiences such as used during remembering thinking about the future, and generally when imagining alternative perspectives and scenarios to the present” (p. 18/19) (Buckner et al., 2008). While the DMN is a good “first approximation” of brain networks necessary for BVSR, it is not sufficient to explain the strong interplay between DMN and other hubs (e.g., semantic networks as implicated in Figure 1 top left) (Lau et al., 2008). We do note that the studies represented in the upper left hand panel of Figure 1 (overlapping the semantic network) represents: (1) higher subject Creative Achievement in normal college subjects (Jung et al., 2010c), higher creative drive (in FTD patients) (Miller et al., 1998), and higher originality scores in lesioned patients (Shamay-Tsoory et al., 2011), thus suggesting some generality of findings across measures of creative cognition with respect to lower brain network integrity. All of these behaviors conform to *sequential BVSR*, involving both exploratory and eliminatory processes, as re-conceptualized by Dean Keith Simonton. And finally, the brain does not “devote” regions to certain cognitive tasks, but rather constantly reuses, co-opts, and optimizes allocation of neural resources toward the demands of ongoing thought processes. Thus, the DMN appears to have been co-opted (or co-evolved) for the purpose of BVSR, with other hubs pulled in as task demands dictate. The production of something “novel and useful” appears to depend, at least in part, on disinhibitory neuronal processes within this core network, while excitatory processes (i.e., more refinement of ideas or *selective retention*) would appear to depend on the CCN. This would be a plausible allocation of cognitive resources with DMN devoted to the innovation and blind variation mechanisms associated with the “constructing of dynamic mental simulations,” while the CCN would be engaged to test retained innovations within the framework of the external environment. This would also be consistent with cognitive research showing a role for dorsolateral prefrontal cortex in “implementation of control” mechanisms, while the anterior cingulate is engaged during performance monitoring (MacDonald et al., 2000). Creative cognition is like other types of cognition, only more specialized in a terms of its focus (i.e., often domain specific) and type of adaptive problem solving (i.e., often abductive as opposed to deductive reasoning).

There is a relatively long and consistent effort to link arousal with creative cognition, with early studies showing decreased creative ideation associated with increased arousal induced by stress (Krop et al., 1969), brainstorming (i.e., social stress) (Lindgren and Lindgren, 1965a,b), and intense white noise (Martindale and Greenough, 1973). This general observation, led to the hypothesis that decreased cortical arousal was associated with increased creative cognition, a notion supported by numerous electroencephalographic (EEG) studies (Fink and Benedek, 2012). For example, increased alpha (a measure of lower cortical arousal) has been associated with creative cognition in highly creative subjects (compared to lower creative) (Martindale and Hines, 1975). Also note that subjects with decreased “latent inhibition” (i.e., the ability to screen from current attention stimuli previously tagged as irrelevant), have been found to have increased creative achievement (Carson et al., 2003), and rely more on intuition in implicit problem solving (Kaufman, 2009). This notion of “disinhibition” of cognitive control mechanisms associated with creativity was first formulated as a “syndrome” by Martindale (Martindale, 1971), with the creative person showing lower levels of frontal inhibition (Martindale, 1989). This notion has great appeal, comports well with the data, and even corresponds well with “folk psychology” notions of the creative process (i.e., the warm bath of Archimedes, the long walks of Beethoven, the dream state of Kekulé, the drug taking (i.e., drinking) of Hemingway, etc.) that all serve to downregulate externally directed cognition and upregulate exploratory idea spaces. Importantly, this “disinhibition” likely corresponds to both BVSR aspect of creative cognition, although the bias would be toward *elaboration*, where multiple ideas can be generated in an environment lacking refinement, formal testing, or selection pressures. Our research tends to support the notion that some normal brains, performing better on standard creativity measures, are more “disinhibited” in their organization, with certain regions having lower cortical volume (Jung et al., 2010c), lower white matter fidelity (Jung et al., 2010a), and (at or below a verbal IQ of 116) anterior cingulate biochemistry tending to “gate” frontal information flow (Jung et al., 2009a). Certain clinical entities (particularly semantic dementia) and lesions within isolated anterior frontal and inferior parietal brain regions support the notion of disinhibitory facilitation of blind variation serving creative cognition (Miller et al., 1998; Shamay-Tsoory et al., 2011; Abraham et al., 2012a). However, other research groups have not found strong evidence to support such a hypothesis of frontal disinhibition subserving blind variation in creative cognition (Takeuchi et al., 2010a,b; Gansler et al., 2011).

Raichle described the discovery of this “default mode of brain function” as a “problem” to the neurosciences in that activity *decreases* were associated with cognition within a discrete network of brain regions (p. 1085) (Raichle and Snyder, 2007). A similar “problem” exists within the cognitive neuroscience of creativity: namely how to account for the growing number of studies showing *decreased* cortical thickness/volume or white matter integrity associated with *increased* human cognitive ability. More neurons and/or dendritic arborization or thicker myelin corresponding to higher levels of cognitive capacity makes intuitive sense. Decreased brain integrity (as commonly understood) associated with higher levels of ability, requires a mechanistic framework by which such relationships can make sense. We shall lay out such a mechanistic framework in a future paper. Suffice it to say that the current results support a disinhibitory bias within a network of brain regions that normally stand in excitatory and inhibitory balance, corresponding to those commonly observed within the DMN (including the anterior-inferior frontal cortex, inferior parietal cortex, and anterior temporal cortex). This DMN normally serves to “instantiates the maintenance of information for interpreting, responding to and even predicting environmental demands (p. 1087).” It is described as a “Bayesian inference engine” designed to make predictions about the future. It can also be viewed here as serving blind variation in creative cognition.

What other evidence do we have supporting network notions of creative cognition? While our purpose was to highlight structural imaging studies, two recent functional imaging studies bears mentioning. Limb and Braun studied six full-time musicians using functional Magnetic Resonance Imaging (fMRI) while they either performed overlearned or improvised piano pieces (Limb and Braun, 2008). They found that spontaneous improvisation was associated with widespread deactivation of the lateral prefrontal cortex along with simultaneous activation of medial frontal cortex, and describe this finding as “intrinsic to the creative process,” being “innovative, internally motivated production of novel material” (p. e1679). In a replication and extension of this work, researchers had rap musicians either perform overlearned lyrics (repeated condition) in the scanner or to create rap on the fly (improvised condition) (Liu et al., 2012). Twelve male freestyle artists were studied. Again, they found a dissociation of activity between brain regions: increased activation within the mPFC and simultaneous decreases within the dorsolateral prefrontal cortex, with subsequent analyses showing concurrent activation within the anterior cingulate gyrus and cingulate motor area. Connectivity analyses found that the mPFC activation was correlated with activations across a broad network including the amygdala, inferior frontal gyrus, and inferior parietal gyrus (p. 5). Thus, this functional study of rap musicians appears to show a back and forth between large brain networks, with improvisation resulting in increased activation of the DMN (and decreased activation within the CCN), as well as modulation of the interplay between these two networks by the salience network (i.e., anterior cingulate, insula, etc.). We hypothesize that the back and forth between these two networks (default, cognitive control) likely corresponds to the BVSR components of creative cognition respectively. While speculative, it would be of interest to determine whether these rap musicians had *decreased* cortical thickness, as compared to age, sex, and IQ matched controls, particularly within inferior parietal and anterior-inferior frontal regions identified in Figure 1.

As others have stated far more adamantly (Dietrich, 2004), creativity is not comprised of one cognitive process, but of many cognitive processes, including (but not limited to) defocused attention, mental flexibility, cognitive control, and other cognitive constructs between the broad ranges of BVSR. The production of something “novel and useful” appears to depend, at least in part, on disinhibitory neuronal processes within this core network, while excitatory processes (i.e., more refinement of ideas or *selective retention*) would appear to depend on the CCN. This would be a plausible allocation of cognitive resources with DMN devoted to the innovation and blind variation mechanisms associated with the “constructing of dynamic mental simulations,” while the CCN would be engaged to test retained innovations within the framework of the external environment. This would also be consistent with cognitive research showing a role for dorsolateral prefrontal cortex in “implementation of control” mechanisms, while the anterior cingulate is engaged during performance monitoring (MacDonald et al., 2000). Creative cognition is like other types of cognition, only more specialized in a terms of its focus (i.e., often domain specific) and type of adaptive problem solving (i.e., often abductive as opposed to deductive reasoning). We believe that future research, focused on disinhibitory hubs within the DMN, will serve to move the study of creativity neuroscience forward in a more focused manner (Buckner et al., 2009). After all, a full understanding of creativity will only be had when we move beyond both folk psychologies and “first approximations” to address this important construct in its full complexity, across domains, species, and timescales.

## Conclusions

1. An appropriate, well-accepted, operational definition exists for creative cognition: *the production of something both novel and useful*.
2. Such a definition can be conceptualized within an evolutionary framework, particularly notions of BVSR.
3. Structural imaging techniques provide a reliable framework within which the field can begin to discuss brain traits associated with creative cognitive abilities.
4. Both increased and decreased brain “fidelity” measures are associated with creative cognitive abilities across a wide array of brain regions.
5. Regions of decreased brain fidelity associated with increased creative cognitive ability tend to correspond to the so-called DMN as demonstrated by case studies, imaging studies, and lesion studies.
6. Focus on cortical hubs within the DMN represents a research opportunity to further refine the manifestation of creative cognition in the brain.
7. The results indicate a dynamic interplay between inhibitory and excitatory networks corresponding to cortical hubs likely corresponding to BVSR components to creative cognition respectively.

### Conflict of interest statement

The authors declare that the research was conducted in the absence of any commercial or financial relationships that could be construed as a potential conflict of interest.

## Footnotes

^1^Although see Nusbaum and Silvia (2011) for relationship of CAQ to Openness/Intellect. Also note that CAQ is heavily weighted toward artistic creativity, especially in college-aged sample.
